# The Study of Composite Materials Properties Based on Polymers and Nano-Additives from Industrial Wastes from Kazakhstan

**DOI:** 10.3390/ma17122959

**Published:** 2024-06-17

**Authors:** Dzhumakhon Sharifov, Rimma Niyazbekova, Avazi Mirzo, Lazzat Shansharova, Mira Serekpayeva, Saule Aldabergenova, Ainur Ibzhanova, Ryszard Machnik, Michał Bembenek

**Affiliations:** 1 “Kazakhstan Institute of Standardization and Metrology” Republican State Enterprise, 11 Mangilik El Ave., Astana 010000, Kazakhstan; shjumm@mail.ru (D.S.); rimma.n60@mail.ru (R.N.); l.sarsenbayevna@gmail.com (L.S.); mika82.02@mail.ru (M.S.); ibzhanova83@mail.ru (A.I.); 2 Scientific Research Institute, Tajik National University, 17 Rudaki Avenue, Dushanbe 734025, Tajikistan; 03-01-90@mail.ru; 3 The Department of Standardization, Metrology and Certification, Technical Faculty, S. Seifullin Kazakh Agrotechnical Research University, 62 Zhenis Ave., Astana 010011, Kazakhstan; ass_1982@mail.ru; 4 Faculty of Mechanical Engineering and Robotics, AGH University of Krakow, A. Mickiewicza 30, 30-059 Krakow, Poland; machnik@agh.edu.pl

**Keywords:** polymer composites, microspheres, microsilica, polystyrene, carbon composite polymer films, absorption spectra

## Abstract

This research is aimed at studying the properties of polymer anticorrosion coatings based on ED-20 resin widely used in practice and industrial wastes. In this work, three basic types of nanoscale nanofillers were chosen: dispersed particles—microsilica, microspheres obtained at Kazakh enterprises, and carbon nanotubes. Physicochemical research methods were used in the research: a laser analyzer for studying the dispersibility of industrial waste and spectrometric research methods. The properties of materials were investigated by standardized methods. The obtained results show that the introduction of microsilica and microspheres obtained at Kazakhstani enterprises, used as additives, improves both the physical and mechanical properties of epoxy composites compared to the standard (control) material. The results of experiments have shown that the optimal content of additives of microsilica and microspheres provides an improvement in the physical and mechanical properties of epoxy composites in comparison with the standard (control) material. Studies have shown that the introduction of microspheres into ED-20 polymer increases impact toughness. The introduction of microsilica into the matrix contributes to the increase of elastic modulus. Experimental studies of optical properties of samples of carbon composite polymer films based on polystyrene (PS) with additives of carbon nanotubes C_60_ and C_70_ and multilayer carbon nanotubes were also carried out. The experimental results obtained for the optical properties of polymer composites based on basic polymers from solid waste and carbon nanotubes showed that the optical properties of polymer composites undergo noticeable changes.

## 1. Introduction

Modern polymer composites are increasingly used in various industries in many countries. Improvement of the functional properties of polymer composites by combining compositions of polymers and various fillers has led to the fact that these materials displace metals and ceramics in traditional areas of their application. Due to the synergistic effect, which is created by combining the necessary characteristics of the individual components of the composite, more advanced materials with improved quality parameters are being developed. At the same time, polymer–nanofiller composites have a wide range of unexplored opportunities for their effective application in technological fields [1,2,3]. The problems of improving anticorrosion properties, strength values, and physical characteristics of polymer composites could be solved by using nanofillers from industrial waste and using polymer matrix from municipal solid waste with nanofillers [4,5,6]. Recycling municipal solid waste solves important ecological problems, in addition to economic ones. Wide possibilities for the application of polymer composite materials in recent years are certainly due to their diversity and inexhaustible variability of their composition, as well as the development of methods for their modification. The primary established methodology for producing polymer composites has been the mechanical mixing of filler and polymer matrix [7,8,9]. One established development course for further advancements of properties of epoxy composites is the introduction of industry-derived technogenic waste into their composition, with the goal of substantially reducing the manufacturing cost of a final product by means of replacing the usage of expensive binders while also gaining different functional properties.

Nowadays, there is a high growth rate of production and wide applications of polymer composite materials in almost all branches of industry, including construction, aircraft and mechanical engineering, electronics, medicine, and power engineering. The technology of creation and the production of polymer composite materials at the nanoscale level has added new advantages and opportunities to this [10,11,12]. Based on the selection of a polymer matrix and the addition of nanoparticles, as well as varying their concentration, new composite materials with desired characteristics can be developed. These new materials with enhanced physical, chemical, and applied properties most often replace or supplement various traditional materials.

The choice of ED-20 in cured form is characterized by high tensile and compressive strength, which is more or less a common property of all epoxy resins, as well as high hardness (scratch resistance), which is not characteristic of all resins. It is prickly when hit hard and has no elasticity of its own. Depending on the task to be solved, the resin can be used both without and with additives that change its properties. The task of studying the properties of micro- and nanodispersions of ASW will allow us to effectively use these wastes to regulate the properties of composite protective coatings. The literature and practical data testify to the great advantages of microspheres and microsilica consisting of silicates [13,14,15]. These are high dispersibility, providing the creation of homogeneous structures, low density, high isotropic compression strength, increased heat resistance, and resistance in aggressive media. Microspheres derived from fly ash have been investigated by various research groups to the present day, and the collective findings have become a basis for utilizing microspheres as a secondary raw material [16]. Microspheres and microsilica are used as fillers for plastics to increase the stiffness and modulus of elasticity of the materials. Microspheres are fine, loose powders consisting of hollow, thin-walled particles of spherical shape, which have aluminosilicates in their composition. They are a light fraction of fly ash. Microsilica consists of spherical-shaped particles, which were formed in the process of purifying waste gases of furnaces in the production of technical silicon. It is therefore considered to be an ultradisperse material. Microspheres enhance the bonding strength between carbon-fiber fabric and resin, which effectively improves the anti-wear and friction-reducing properties of fractional materials [17,18]. The application of coatings is associated with certain problems, such as low strength under impact loads and cracking. Therefore, the development of new compositions of coatings based on epoxy resin and industrial waste, such as microsilica and microspheres, is an urgent task that will contribute to improving the characteristics of coatings.

As a rule, the selection of a polymer matrix and nanoparticles (filler) for subsequent production of a future new composite polymer material is based on proven scientific methods and technology. In the first stage, the main purposes of the involved components—polymer matrix (in the role of binder) and nanofiller—are pre-determined. The polymer matrix binds the filler to each other, thus ensuring that all participating components (or particles, if a dispersed filler is used) work together, creating a monolithic of the future material and stress transfer (distribution). Therefore, many physical and chemical characteristics of future samples practically have the reliance on initial binder properties [19,20]. The main purpose of the filler is to strengthen the matrix to give the material the special properties required in advance while also decreasing the cost of raw materials and energy consumption during production. The introduction of various nanoscale particles (nanotubes, fullerenes of different modifications—C_60_, C_70_, fullerene soot, and niello—carbon nanodiamond particles, graphene, carbon fibers, highly dispersed graphite, carbon black, and others) into the matrices of polymeric materials as fillers and reinforcing components is of great scientific and practical interest [21,22,23]. A special place is occupied by industrial wastes containing particles of nanoscale range—microspheres and microsilica. Thus, by adding nanoparticles of a particular type in given percentage proportions in polymeric material matrices, it is possible to obtain new composite polymeric materials and films due to their specific characteristics and dimensions. By varying the combinations between the components involved or, for example, the mass concentration of carbon nanoparticles, it is thus possible to obtain composite materials having the desired properties and the desired set of performance characteristics. The polymer-based carbon composite materials thus obtained combine the qualities of the constituent components: flexibility, elasticity, machinability, hardness, resistance to wear, high light refractive index, and the like. This combination improves many of their performance properties compared to the original components. The available extensive literature shows that many of their physical and chemical characteristics can be significantly, sometimes many times, improved by their modification with small additives of micro- and nanoparticles, carbon nanoparticles—fullerenes, nanotubes, nanofibers, and other inorganic nanoparticles [24]. Therefore, as the analysis shows, the creation and study of nano-containing, carbon-containing polymer composite materials is the most urgent and promising task, the solution of which contributes to the improvement of technological and operational properties of materials for various purposes. Fundamental interest in the object using nanotubes is due, first of all, to its unusual structure and a large array of changes in both chemical and physical characteristics depending on chirality. Many of these properties are still today the subject of intensive research aimed at revealing new interesting features of nanotube behavior in one or another situation [25]. As noted in [26,27,28], PS was among the initial polymers that have been successfully altered by carbon nanoparticles (fullerene) using varying methods. The mechanisms of producing carbon composite materials based on PS can be different: the processes of radical and anionic polymerization, the preparation of appropriate compositions, and the polymerization of styrene in the presence of fullerene, both in the block and in an aromatic solvent with the formation of dark brown composite material and others [29]. The use of the modification of nanocarbon particles—fullerenes C_60_, C_70_, and MWCNT—as a nano-additive is justified by the fact that they have a molecular structure (unlike two other allotropic forms of carbon—graphite and diamond—which have a crystalline structure and represent a periodic lattice of atoms) and are relatively effective modifiers of polymer matrices of various nature [30]. The modifying ability of fullerene is due to the unusual chemical and electronic structure of carbon nanoparticle molecules, which have a nanometer size (diameter C_60_ of the order of 1 nm), a high value of electron affinity (2.7 eV for C_60_), and a comparability of the magnitude of the local free volume fluctuation in some polymers with the volume of the nanoparticle molecule [31]. Therefore, fullerene-containing polymers, as noted, for example [30], can be obtained by isolating composites from an organic solvent solution in which both components (both polymer and fullerene) are well dissolved. It can be assumed that in the case of achieving molecular distribution of fullerene in a continuous polymer phase, direct contact of the macromolecules of the matrix with fullerene molecules is possible.

Therefore, as the analysis shows, the creation and study of nanoscale, carbon-containing polymer composite materials is the most urgent and promising task, the solution of which contributes to the improvement of technological and operational properties of materials for various purposes. This research aims to investigate the properties of polymer compositions using industrial waste from Kazakhstan containing nanodispersions and polymer films with nanofillers. Spectrophotometric methods were used in the studies, which provide essential data with regard to the transmittance, absorption, and reflectance spectra of the studied samples [32,33,34].

## 2. Materials and Methods

Experimental studies of optical properties (absorption, reflection, transmission, and forbidden zone energy spectra) of some samples of carbon composite polymer films based on polystyrene (PS) have also been carried out. The experiments included studies of the element composition of industrial wastes—microspheres and microsilica, phase composition, and particle sizes. Mechanical properties were determined for composites with different contents of dispersions. Experimental studies of polymer films based on the base polymer polystyrene included studies of optical properties (absorption, reflection, transmission, band gap energy spectra) of carbon composite polymer films with carbon nanotube additives. Optical properties affect the variation of important characteristics of dielectric properties of materials.

Experimental studies of epoxy composites with technogenic wastes of the enterprises of the Republic of Kazakhstan have been conducted. The properties of wastes containing nanodispersions of microsilica and microspheres have been investigated. The properties of epoxy resin composites have been studied by means of physical and chemical analysis methods. Investigations of optical properties included the study of absorption, reflection, transmission, and forbidden zone energy spectra of some samples of carbon composite polymer films based on polystyrene (PS) with carbon nanotube additives. Experimentally, the optical absorption spectra (reflection and transmission) of all samples were measured using a Cary—60 UV-Vis spectrophotometer (Agilent Technologies, Santa Clara, CA, USA) in the range from 190 to 1100 nm.

### 2.1. Materials

#### 2.1.1. Materials for Epoxy Resin Composites

The experiments also involved investigating the characteristics of polymer composite materials derived from epoxy resin ED-20, widely used as a coating for structural materials, and poorly studied multi-tonnage waste—microspheres from the ash of the poorly studied Kazakhstani coals of Ekibastuz basin. Also, properties of materials derived from epoxy resin ED-20 and microsilica—a waste product of ferroalloy production in Kazakhstan—were studied for the first time as a filler. Elemental, granulometric compositions of wastes were studied. The properties and structures of composites with additives of microsilica and microspheres were investigated by means of IR spectroscopy and SEM methods. The mechanical properties of composites with different contents of dispersions were determined using standard methods.

ED-20 epoxy resin (GOST 10587-84) (Scientific and Production Company “Astat”, Dzerzhinsk, Russia) was utilized as a binding agent in the produced compositions. While having a resistance to high temperatures, ED-20 is known for its distinct brittleness; the effects of this can be mitigated by adding specific modifiers and hardening agents into polymer blends. Polyethylene polyamine (PEPA) (TU 2413-010-75678843-2012) (Scientific and Production Company “Astat”, Dzerzhinsk, Russia) has been utilized as a hardening agent. Its purpose is to cure epoxy resins at room or low temperatures, even under high humidity conditions. The hardening agent can be described as a liquid with a color profile ranging from light yellow to dark brown and free from any physical impurities.

Waste materials produced in Kazakhstan include microspheres (Public district power plant, Ekibastuz, Kazakhstan) and microsilica (“Tau Ken Temir” silicon plant, Karaganda, Kazakhstan), which were utilized in polymer compositions. Microspheres can be characterized as hollow, tiny solid particles that are a by-product of coal burned in power plants, emerging from the bottom ash. The formation of microsilica is a process that occurs during the smelting of silicon alloys (ferrosilicon). Following oxidation and condensation processes, a portion of silicon monoxide transforms into an ultra-fine product featuring spherical particles and increased amorphous silica content.

#### 2.1.2. Materials for Polystyrene-Based Composites

Carbon nanoparticles of various modifications, fullerenes, were selected as added components in the PS matrices: C_60_ (with concentrations of 1, 3, 5, and 10 wt%), C_70_ (with concentrations of 1.3 and 5 wt%), and multilayer carbon nanotubes (MWCNT with concentrations of 0.1 and 0.5 wt%) (Scientific and Production Company “Astat”, Dzerzhinsk, Russia).

The choice of polystyrene as the matrix of the starting material is because PS is the most well-known and widely used polymer material with several excellent properties, including thermal stability and mechanical and optical properties.

The studied samples were polystyrene films, and composites were based on it with the addition of carbon nanoparticles of fullerenes C_60_, C_70,_ and MWCNT with mass concentrations from 0.1% to 10% of different thicknesses (Table 1).

**Figure 1:**
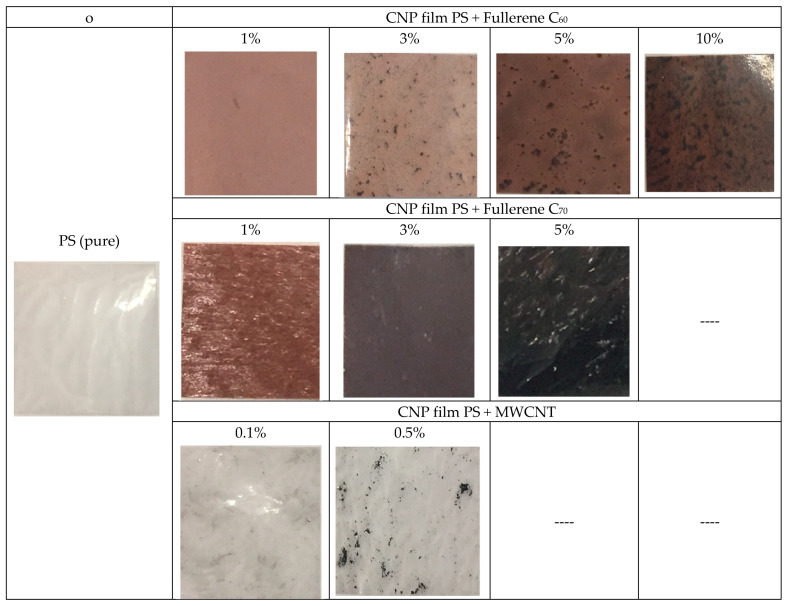
The 10 × 10 mm size images of the samples.

PS films cast from solutions in bromobenzene have high mechanical properties. However, when a filler with the same size of nanoparticles (i.e., fullerene molecules) is introduced into them, the mechanical behavior of the films does not change. It should be particularly noted that the samples obtained are based on MWCNT. This form of carbon, in its structure, occupies an intermediate position between graphite and fullerenes. However, many properties of carbon nanotubes have nothing to do with either graphite or fullerenes. This makes it possible to consider and study nanotubes as an independent material with unique physicochemical characteristics. Therefore, the study of the effect of carbon nanotubes as a nano-additive in PS matrices is of significant fundamental and applied interest. Figure 1 shows the appearance (photo illustration) of the studied samples of carbon composite polymer (CNP) films based on PS and the average values of their thickness, which ranged from 30 to 110 µm. All film samples (Figure 1) prepared for spectrophotometric analysis had the shape of squares with a size of 10 mm.

### 2.2. Methods

Under the following regimes, epoxy compositions filled with microspheres and microsilica were prepared:

- ED-20 pre-dosing;
- Microspheres and microsilica dosing as filler and their further addition to epoxy oligomer in the amount of 2, 5, 10, 15, 20 wt%;
- adding PEPA in the ratio of resin:hardener (1:10) and then mixing the composition

The prepared mixture was filled into molds in the form of 4 × 10 × 80 mm bars and allowed to harden for 24 h at 20–25 °C. The control sample was made without the addition of filler. The test specimens were cast in the form of spatulas for tensile strength testing.

After curing, they were dried in an oven (drying oven-80-01MK SPU) in the following conditions: τ = 24 ± 0.1 N, 1 h at T = 90 °C; 1 h at 120 °C; then slowly cooled to room temperature. These experiments were conducted using mathematical methods of experiment planning with several experiments equal to 12 and a two-factor plan with the number of experiments equal to 9.

The elemental composition was analyzed using an X-ray fluorescence spectrometer Epsilon 1 (Malvern Panalytical, Malvern, UK). The particle size of the fillers was determined on a Mastersizer 3000 laser granulometer (Malvern Instruments Limited, Malvern Worcestershire, UK) with a HydroMV nozzle (120 mL) using water as the dispersion medium. The filler phase composition was determined by X-ray diffraction on a smartLAB X-ray diffractometer manufactured by Rigaku Corporation (The Woodlands, TX, USA). The source of X-ray radiation was a CuKa (1.54059) X-ray tube. The filler microstructure was investigated by scanning electron microscopy TM3030 (Hitachi, Tokyo, Japan). The tube current and voltage were set to 50 mA and 40 kV, respectively. A one-dimensional detector D/teXUltra (Rigaku, Tokyo, Japan) with a Kß filter was used. Measurements were performed using a step-scanning method with a step width (δ 2 θ) = 0.1°, scanning speed = 2 deg/min, and measurement angle range of 2 θ = 5–90°. Mechanical tests of polymer composites were performed on specimens in the form of a double-sided blade. In this way, tensile fracture stress, tensile and bending moduli, and compressive fracture stress were tested. The experiments were realized on the universal testing machine WDW-5E. The impact toughness of the samples was determined on a pendulum impact bench KMM-M (TechnoKazakhstan LLP, Astana, Kazakhstan) according to the Charpy method.

The absorption spectra of the samples (reflection and transmission) were measured using modern spectrophotometric devices: the V-770 Spectrophotometer (Jasco, Tokyo, Japan), as well as the Carry-60 UV-Vis spectrophotometer (Agilent Technologies, Santa Clara, CA, USA) in a range of wavelengths from 200 to 800 nm.

As a rule, spectrophotometry (or absorption spectroscopy) is a physicochemical method for qualitative and quantitative analysis of substances based on measuring absorption spectra in the optical region of electromagnetic radiation in the wavelength range 190 ÷ 1000 (nm). This wavelength range corresponds to near UV (λ ≈ 190 ÷ 400 nm), visible region (λ ≈ 400 ÷ 760 nm), and near IR (λ ≈ 760 ÷ 1000 nm). The basic law that underlies the operating principles of spectrophotometric instruments is usually the Bouguer–Lambert law:

(1) $$I=I_{0}e^{-\alpha (\lambda )d},$$

where α(λ) is the absorption coefficient and, as a rule, is different for different wavelengths and does not depend on the intensity of the incident light and the thickness of the absorbing layer d. An analysis of the spectral composition of optical radiation makes it possible to determine a number of important physicochemical parameters [28]:

- the atomic, molecular, and crystal structure of various substances and their optical properties;
- the structure of polymers and their isotropic and isomeric modifications, as well as the structure of intermediate formations, such as chemical radicals and molecular associations;
- the chemical composition of an unknown sample by the presence of certain spectral lines (or groups of lines) and their intensity;
- the band gap energy, etc.

The absorption coefficient α(λ), where λ is the wavelength of optical radiation, can be evaluated by the optical absorption spectrum A(λ) (absorptivity) with the expression [35]:

(2) $$\alpha \left(\lambda \right)=2.303\frac{A(\lambda )}{d}$$

where d is the thickness of the studied samples (film) in cm, and the value of A(λ) for the corresponding wavelength can be determined as the logarithm of the ratio of the intensity of the incident I_0_ (λ) and the transmitted I(λ) beam, i.e., as A(λ) = log I_0_ (λ) ⁄ I(λ).

Absorption spectra, and more particularly the absorption edge, are a valuable method for studying the optically induced transition and obtaining information about band structure, binding energy, and energy (width) band gaps. For instance, the absorption edge for direct and indirect transitions can be obtained by taking into account the models proposed by Tauc et al. [22,28]:

(3) $$\alpha \times E_{F}=C_{0}{\left(E_{F}-E_{g}\right)}^{n}$$

where E_F_ = hυ is the photon energy (h is Planck’s constant, υ is the frequency), α is the absorption coefficient, C_0_ is an energy-independent constant related to the properties of the valence and conduction bands, E_g_ is the band gap energy of the sample under study, n = 1/2; 3/2; 2 or 3—for direct allowed, direct forbidden, indirectly allowed and indirectly forbidden transitions, respectively.

**Figure 2:**
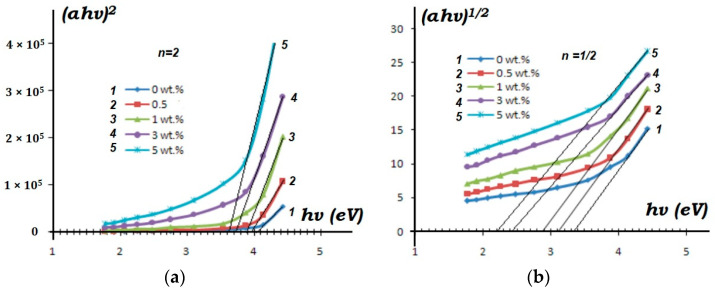
Tauck graphs of ZnO-co-doped PS thin films for different dopant concentrations with energy gap: (**a**) allowed direct transitions; (**b**) allowed indirect transitions [27].

To determine the band gap energy E_g_ from the absorption spectrum for direct allowed transitions (E_F_ ≥ E_g_), it is necessary to plot the dependence of the (α×E_F_)^2^/f(E_F_) and select a linear section on it. Extrapolating it to the intersection with the *x*-axis, one can determine E_g_. For example, band gap energies were determined for polystyrene (PS) thin films with different concentrations (0, 0.5, 1, 3, and 5 wt%) of zinc oxide (ZnO) nanoparticles by using this method (Figure 2) [27].

The extinction coefficient k is related to the optical absorption coefficient α(λ) by the dependence:

(4) $$k=\frac{\alpha \left(\lambda \right)\times \lambda }{4\pi }$$

From the value of normal reflectivity R, the refractive index n of the samples can be determined [36]:

(5) $$n=\left[\frac{1+R}{1-R}\right]+\sqrt{\frac{4R}{{\left(1-R\right)}^{2}}-k^{2}\;}$$

## 3. Results and Discussion

### 3.1. Epoxy Resin-Based Materials

The results of the determination of elemental composition showed that microsilica mainly consists of oxides of Si, Ca, Fe, and K and also contains small admixtures of oxides of Mn and Mg.

**Figure 3:**
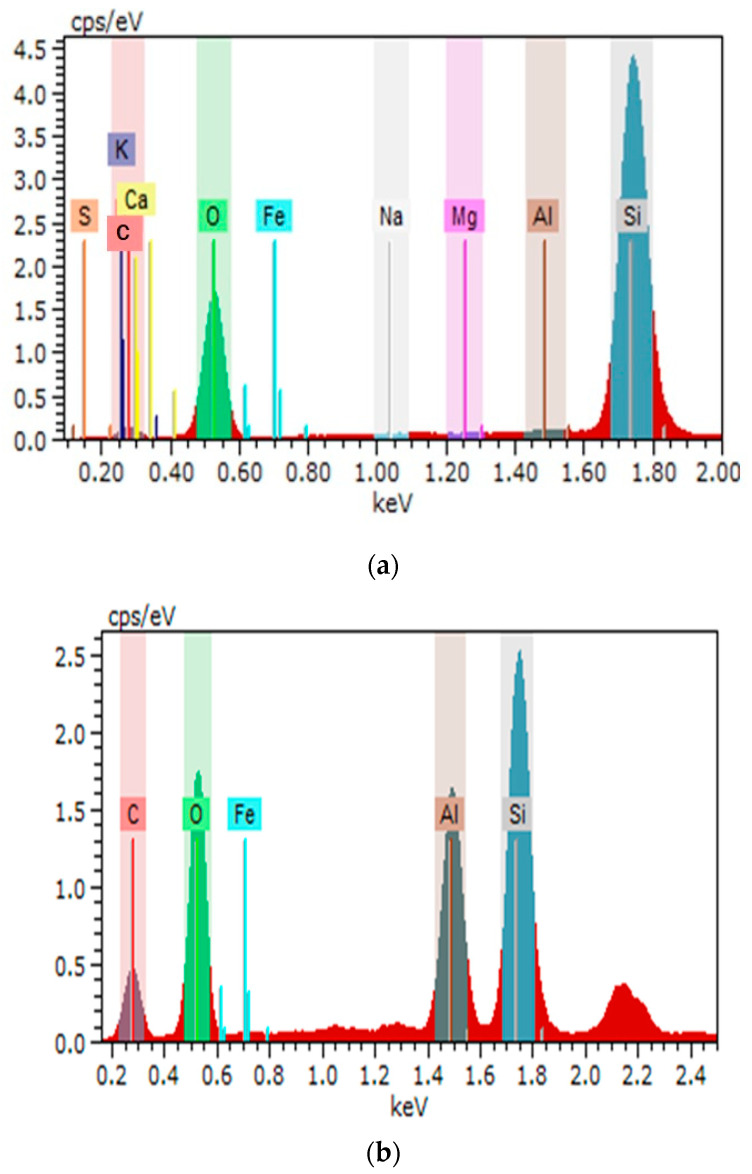
Elemental analysis of (**a**) microsilica; and (**b**) microspheres.

The studies of the microsphere chemical composition indicated their high content of Al_2_O_3_—13.2%, SiO_2_—23.7%, and Fe_2_O_3_—3.1%, and they also contain small impurities of oxides P, K, Ti, Na, and Ca (Table 2) (Figure 3).

Figure 3a,b shows the distributions of chemical elements on the surface of microsilica and microspheres obtained by energy dispersive analysis.

**Figure 4:**
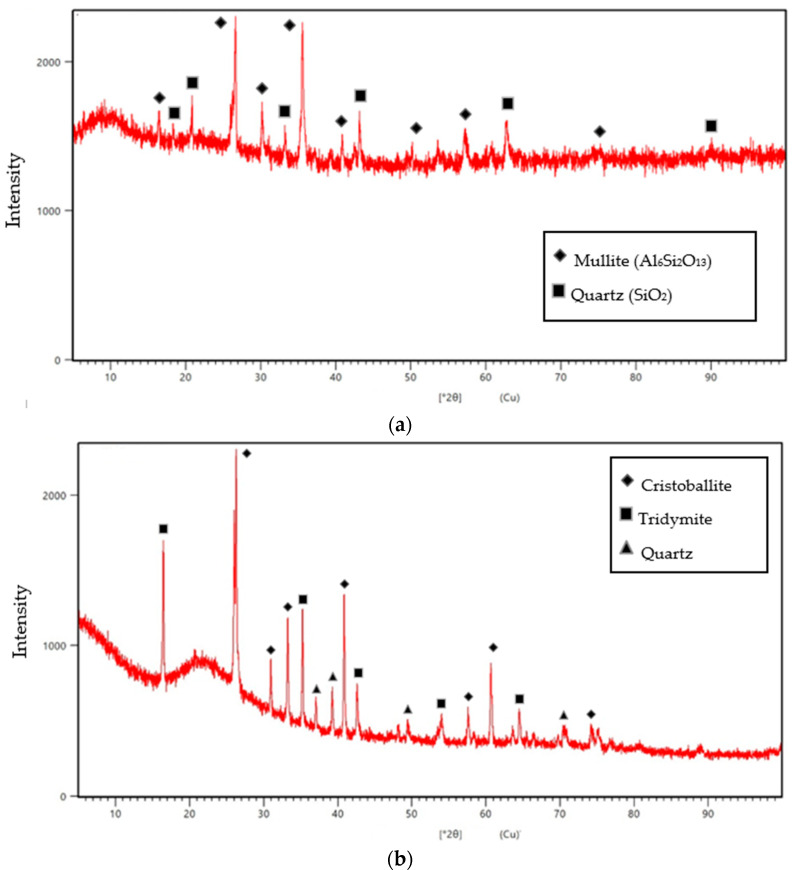
Phase composition of (**a**) microspheres; and (**b**) microsilica.

The peculiarities of the processes of structure formation of composites containing dispersions of industrial wastes have been revealed by X-ray method structure analysis. Figure 4 shows the microsphere and microsilica phase compositions.

According to X-ray method structure analysis, the microspheres are represented by a mixture of mullite and quartz phases. The phase composition of microspheres determines their morphology; samples with a high content of X-ray amorphous phase are formed in the form of thin-walled cenospheres, and samples containing mullite phase are characterized by relief and perforated shell morphology (Figure 4a). In phase structure studies of microsilica, the formation of cristobalite, tridymite, and α,β—quartz is observed. Each of these phases, a polymorphic modification, is stable in a certain region of temperatures and pressures. Silicon dioxide has a complex polymorphism, which at normal pressure has the following type of transformations occurring upon heating: quartz–tridimite–cristobelite (Figure 4b).

The effectiveness was established, quantitative dependencies of the change in strength properties on the amount and size of dispersions were revealed, and effective compositions were developed.

**Figure 5:**
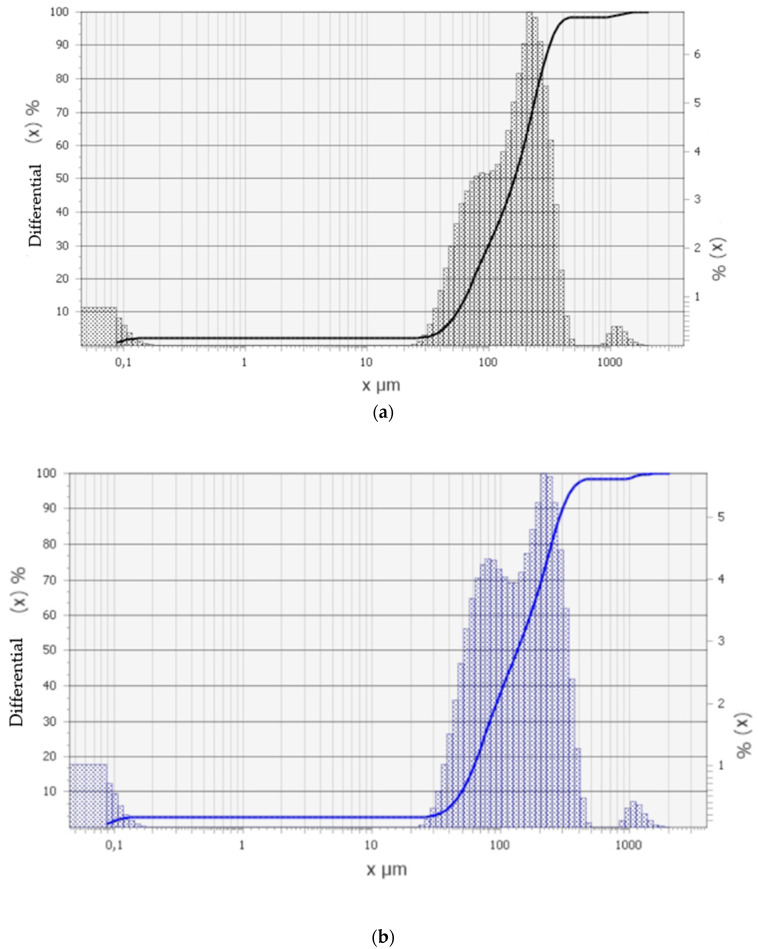
Particle size distribution: (**a**) microspheres sample; (**b**) microsilica sample.

The main component of microsilica is amorphous silicon dioxide. The particle size distribution is shown in Figure 5. 

Microsilica and microsphere fractional content can be described as having bimodal distribution, and the particle size range is observed to be from 0.1 to 1000 μm. The average particle sizes are approximately 30 μm and 300 μm. Mechanical tensile tests of polymer compositions are important for applicable industries, so their values and modulus of elasticity are given in Table 3.

Impact toughness tests were conducted to ensure the quality of the samples, as well as to assess the response of polymer composites to impact stress. Therefore, the brittleness and toughness of samples could be evaluated. The results of impact toughness tests are summarized in Table 4.

Quantitative dependencies of changes in the composite physical properties with microsphere additives were obtained. The compositions of polymer composites based on ED-20 have been optimized by the indices of strength and chemical resistance. The effects of size and number of filling particles on the characteristics of cured composites modified by additives of microspheres and microsilica have been investigated using the methods of mathematical planning of experiments. The optimal compositions of filled composites in terms of strength parameters have been established. Observing the results of microsphere addition, samples with less than 5% microsphere additives showed a 9% increase in tensile strength. The addition of more than 5% microspheres did not produce any significant difference.

**Figure 6:**
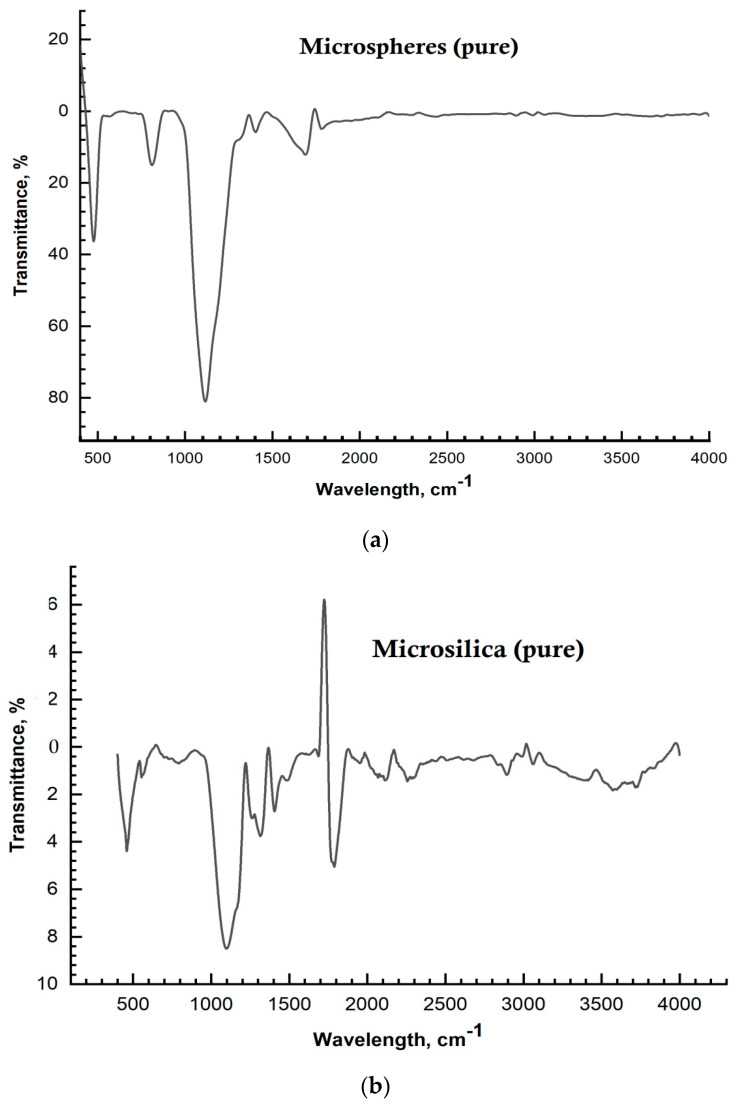
IR spectra: (**a**) microspheres; (**b**) microsilica.

In the IR spectrogram of the initial microsilica, the absorption bands in the region of 470–1120 cm^−1^ are characteristic of the vibrations of the SiO^4−^ group, and the bands of 1118 cm^−1^, 805 cm^−1^, and 480 cm^−1^ correspond to the deformation vibrations of Si-O of amorphous silica and the Si-O-Si bond (Figure 6a).

In the IR spectrogram of the initial microspheres, the absorption bands in the region of 1093, 12 cm^−1^ are characteristic of (Si, Al)-O. Aluminum oxide makes an intensive contribution to the formation of the structure of microspheres (Figure 6b).

**Figure 7:**
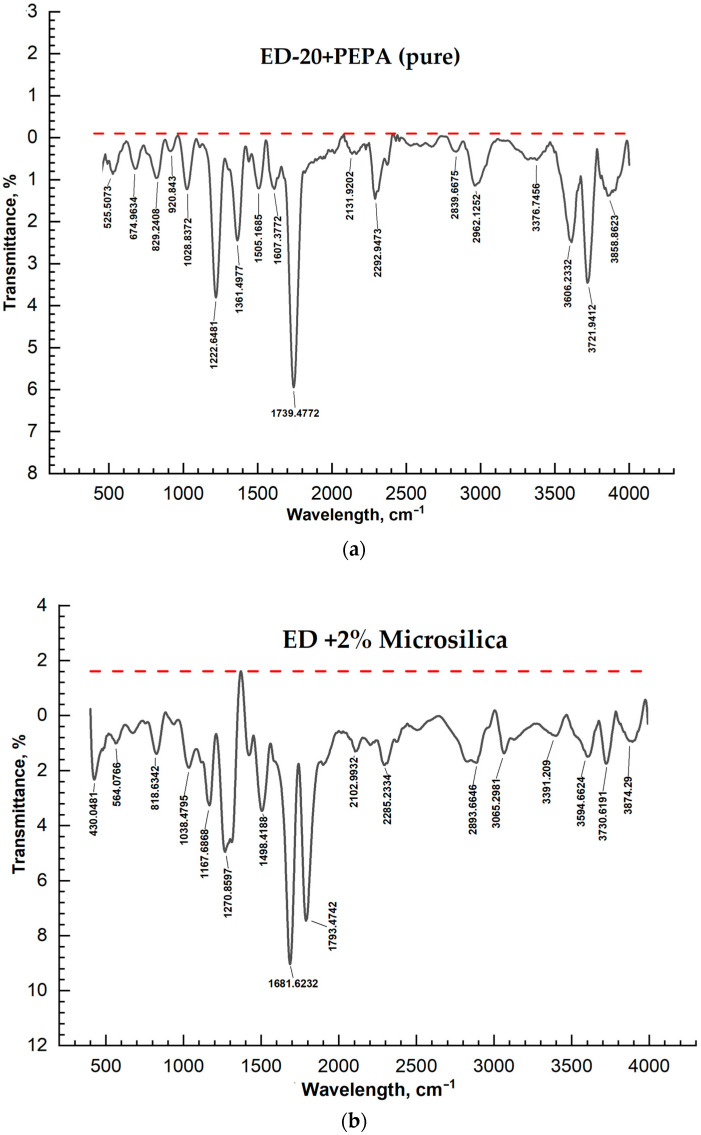

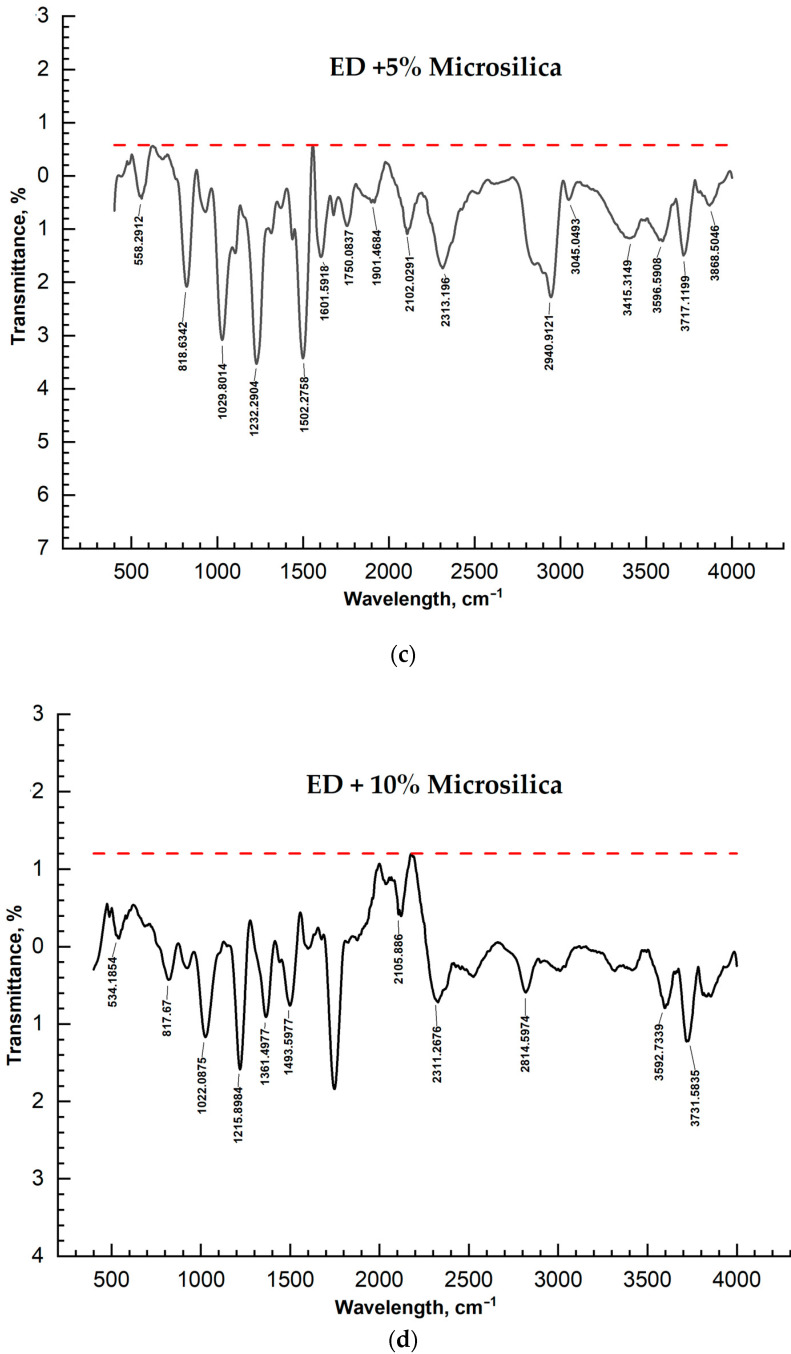
IR spectra: (**a**) ED-20 + PEPA; (**b**) ED-20 + PEPA + 2% microsilica; (**c**) ED-20 + PEPA + 5% microsilica; (**d**) ED-20 + PEPA + 10% microsilica.

**Figure 8:**
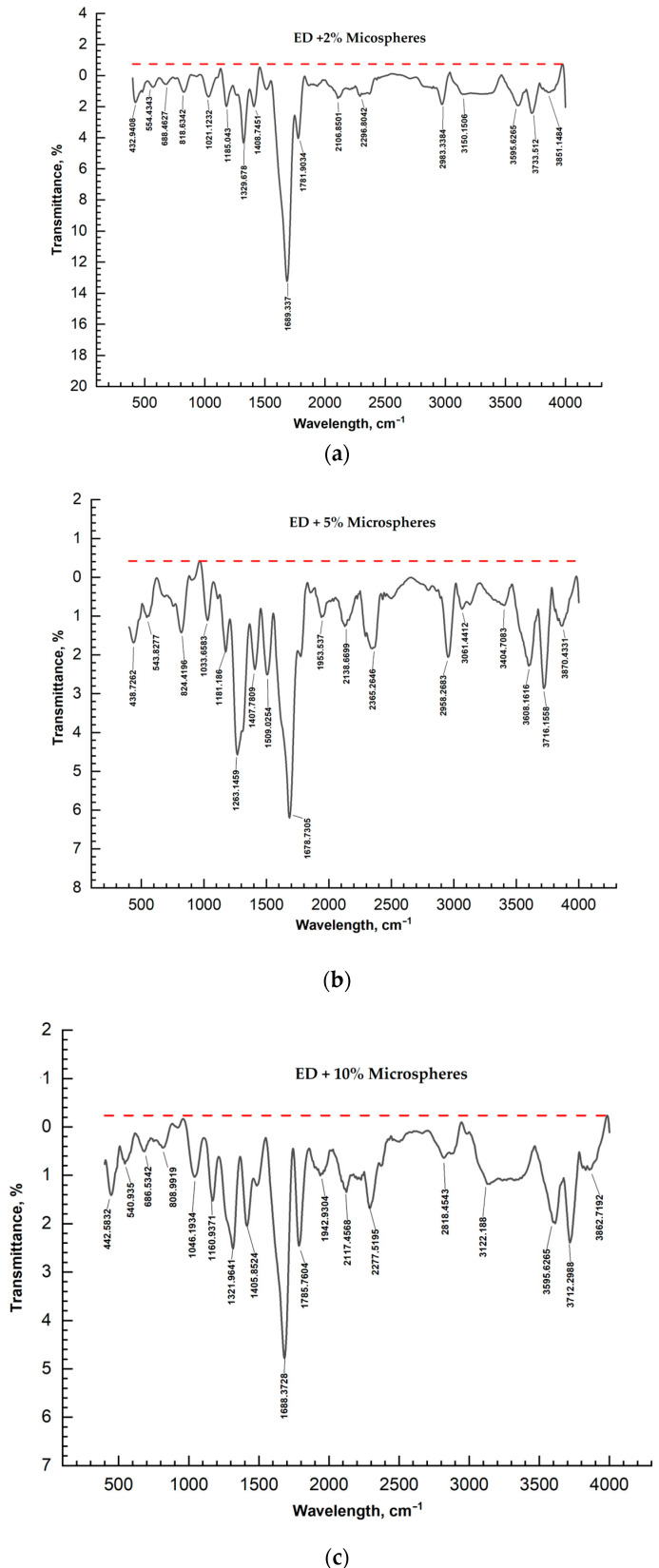
IR spectra: (**a**) ED-20 + PEPA + 2% microspheres; (**b**) ED-20 + PEPA + 5% microspheres; (**c**) ED-20 + PEPA + 10% microspheres.

The IR spectra of pure ED-20 samples and samples with added microsilica and microspheres with mass concentrations from 2 to 10% were studied using an FT/IR-4700 FT/IR spectrometer (Jasco Japan) in the wavelength range from 400 to 4000 nm. The results of measurements of IR spectra of the samples are shown in the figures below (Figure 7 and Figure 8).

Analysis of the IR spectrum confirms the presence of characteristic bands 1028–1167 (valence vibrations of C-O) and 1120–1500 cm^−1^ (deformation vibrations of C-H) (Figure 6). A comparison of the results of the IR spectra indicates that the changes in the peak intensities observed in the IR spectrum are caused by the influence of microsilica particles. The microsilica particles introduce perturbations in PEPA corresponding to the wave number interval 1120–1500 cm^−1^. The presence of different characteristics of the curing process in the curing resin can also be judged by the absorption bands at 2285–2313 cm^−1^ (valence vibrations of triple bonds C=C, C=N), as well as by the absorption of primary and secondary amino groups. The intensity of two absorption bands 3065–3200 cm^−1^, characteristic of the amine group RNH, decreases with an increasing amount of microsilica, which indicates the presence of associated groups due to the formation of hydrogen bonds between them.

A somewhat different picture is observed when microspheres are used. On the IR spectra, the intensity of the peaks of samples 13,990–1400 cm^−1^, characteristic of valence vibrations of C-O and deformation vibrations of C-H and C=C, increases as the number of microspheres increases. Due to the fact that deformation vibrations of silicate groups of microspheres lie in this frequency range, the effect of resonance of frequencies of natural vibrations of microsphere particles and deformation vibrations of C-H and C=C is possible. The intensity of the absorption band at 3100 cm^−1^, characteristic of the amine group R_2_NH, does not change linearly with an increasing number of microspheres. The growth of peak intensities taking place in the IR spectrum is a result of the influence of microsphere particles. Microspheres introduce perturbations corresponding to the interval of wave numbers 3100 cm^−1^, probably due to the inhomogeneity of microsphere sizes as well as surface defects. The highest intensities are characteristic when using from 2 to 5% microspheres. Figure 8 shows the structure of the used microspheres, which are hollow silicate spheres. As can be seen from Figure 8, the structure of the cured mixture changes significantly when 5% microspheres are introduced; some particles are deformed. This fact probably influences the curing, hardening, and chemical stability of the composite. The use of modifiers compatible with oligomer has an effect on filled epoxy compositions because, apparently, the interaction at the interface is reduced, the conditions of contact between binder and filler are improved, and the processing of the composition is improved. The effect of fillers on the structure formation processes of epoxy compositions is very ambiguous, which is largely due to the different activities of fillers. The study of curing kinetics showed that the introduction of 2 wt% of microsilica already improves the process of structure formation, which is confirmed by the results of determining physical and mechanical properties. Microspheres, similarly to microsilica, accelerate the process of structure formation, which is influenced by the fractional composition and defectiveness of their surface. The introduction of 5% and higher microspheres intensifies the process of structure formation.

**Figure 9:**
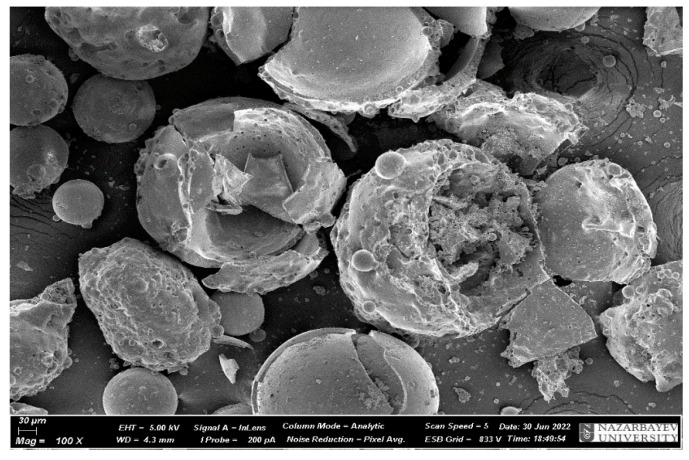
Microphotographs of microsphere samples.

**Figure 10:**
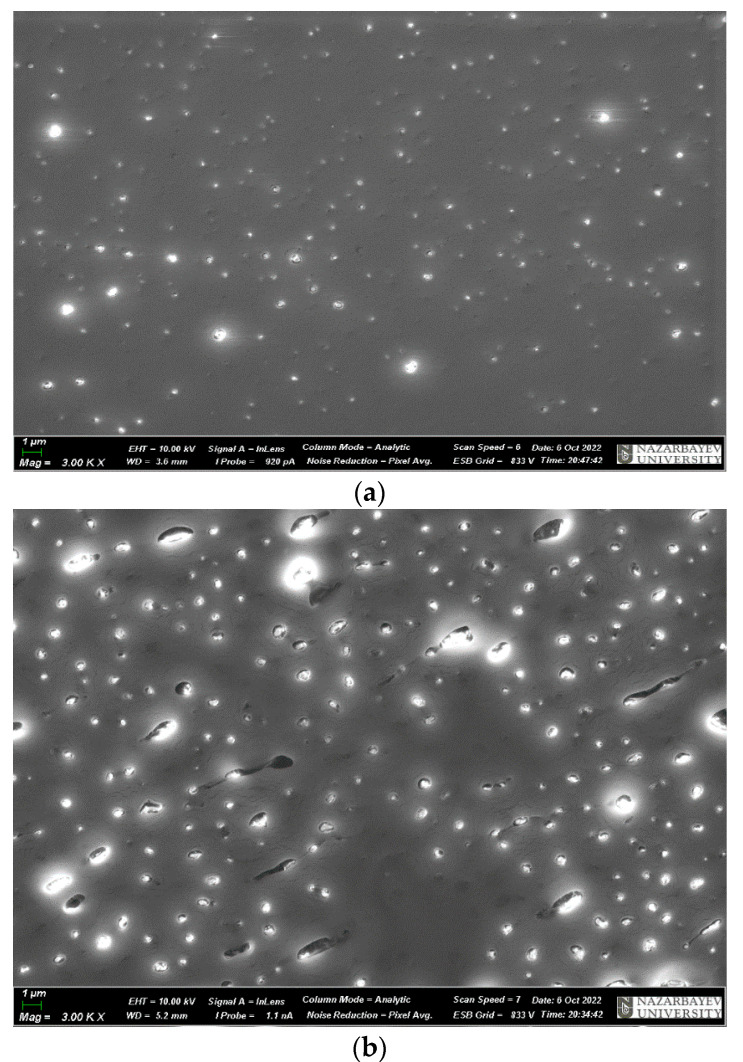

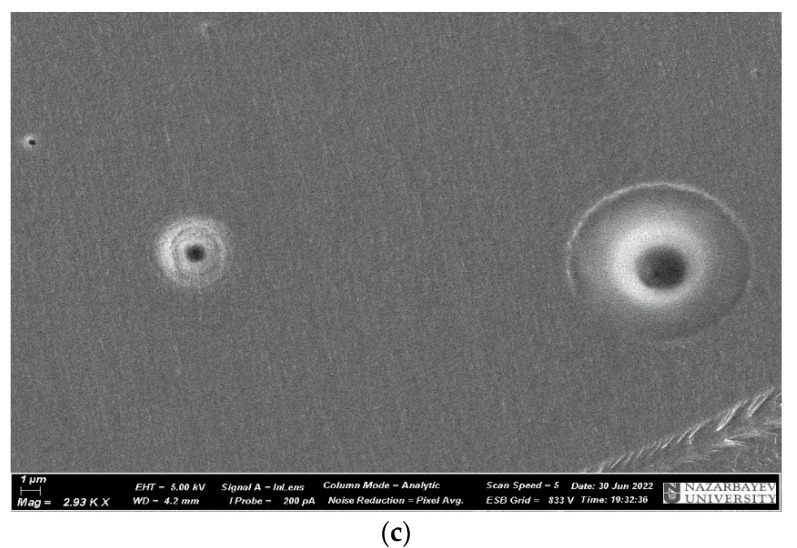
Microphotographs of samples of ED-20 + PEPA mixture with microsphere additives after curing after 1 h: (**a**) ED-20 + 2% microspheres; (**b**) ED-20 + 5% microspheres: (**c**) ED-20 + 10% microspheres.

Figure 9 and Figure 10 show microphotographs of microsphere samples and samples of ED-20 + PEPA mixture with microsphere additives.

**Figure 11:**
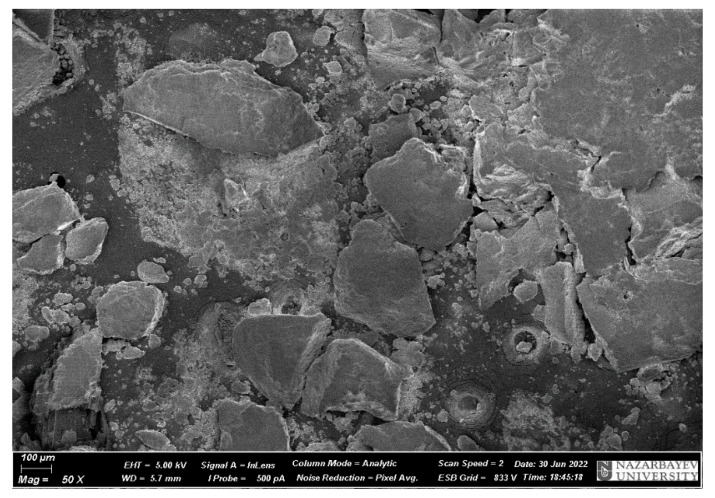
Microphotographs of microsilica samples.

In Figure 11, microphotographs of samples with added microsilica, which represents micro- and nanoparticles, are presented.

The results of electron microscopic analysis of the microsilica powder are shown in Figure 11. Microscopic analysis shows the presence of the pronounced agglomeration of particles, with the size of agglomerates from 5 to 100 microns. Agglomerates have a dense, homogeneous structure.

**Figure 12:**
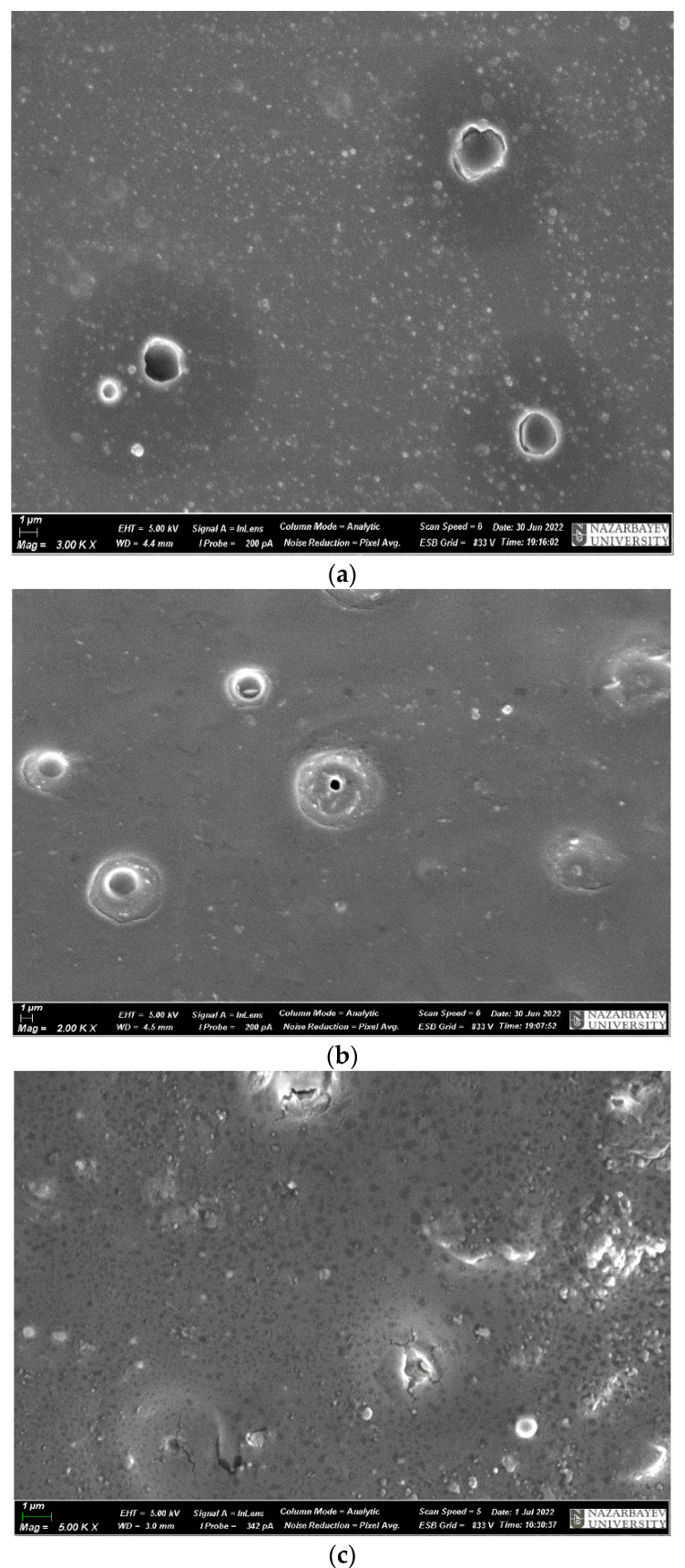
Microphotographs of samples of ED-20 + PEPA mixture with microsilica additives after curing after 1 h: (**a**) ED-20 + 2% microsilica; (**b**) ED-20 + 5% microsilica; (**c**) ED-20 + 10% microsilica.

Figure 12 shows micrographs of samples of a mixture of ED-20 and microsilica.

Microphotographs of the samples show that the surfaces of the chips of the samples in Figure 12a–c have light areas, which are due to the presence of a silica component in the epoxy matrix. It should be noted that the amount of silicon in the composite structure is less than it should be for fully hydrolyzed microsilica. This indicates that the microsilica particles are defective due to the presence of a large number of residual hydroxyl groups on their surface. This gives reason to attribute such a structure formation to globules formed around microsilica particles. It can be assumed that transitional interpenetrating layers are formed between the silicate particles and the polymer matrix. Depending on the amount of filler, such globules are formed throughout the entire volume of the composite. When using microsilica in polymer compositions, thousands of microparticles compact the polymer mixture, which improves the adhesion to the matrix. As a result, microsilica, already at an addition of 2%, contributes to a stronger composite structure and improved chemical resistance, which can be seen in Table 3. Some decrease in strength properties is observed when the additive is increased up to 10%. As a result of the occurrence of agglomerates, microcracks, and microstresses, which can be seen in Figure 12, some decrease in impact strength is observed.

The obtained results hint that the more optimal composition of additives is 5 wt% of microspheres and 5% of microsilica, as they produce improved physical and mechanical properties: the ultimate strength increased by 7%, and the elasticity modulus of specimens with the addition of microspheres increased by 19%.

The addition of 10% microspheres gave better impact strength values.

Experimental observations show that satisfactory physical and mechanical properties of composite materials are achieved through adding microspherical filler to the epoxy polymer.

The possibility of using microspheres and microsilica for obtaining materials with improved physical and technical properties has been experimentally confirmed. The obtained results allow for solving economic and ecological problems associated with the production of polymer composites.

Efficiency has been established, quantitative dependencies of change of strength properties on quantity and sizes of dispersions have been revealed, and effective compositions have been developed.

### 3.2. PS (Polystyrene)-Based Materials

**Figure 13:**
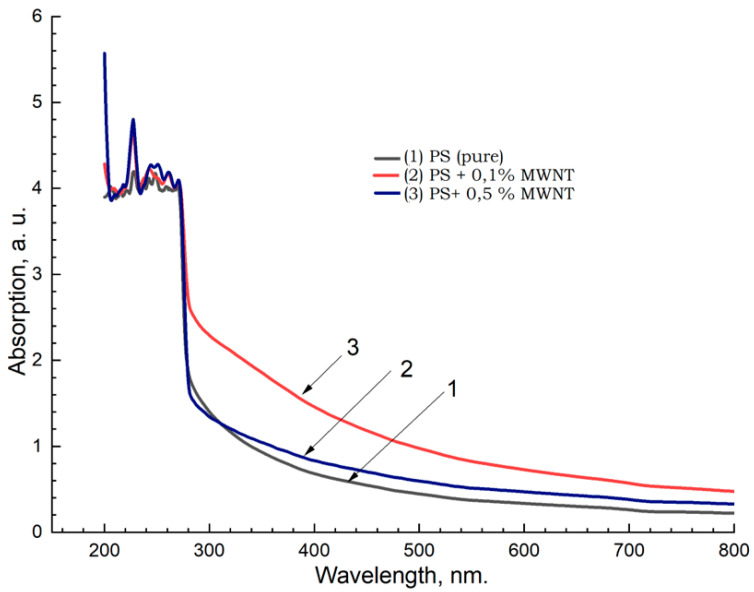
Absorption spectra of PS + MWCNT samples: 1—PS pure; 2—PS + 0.1% MWCNT; 3—PS + 0.5%MWNT.

The results of spectrophotometric studies of PS + MWCNT, PS + Fullerene C_60,_ and PS + C_70_ samples, i.e., spectra of (a) absorption, (b) reflection, and (c) transmission, are shown in Figure 13.

**Figure 14:**
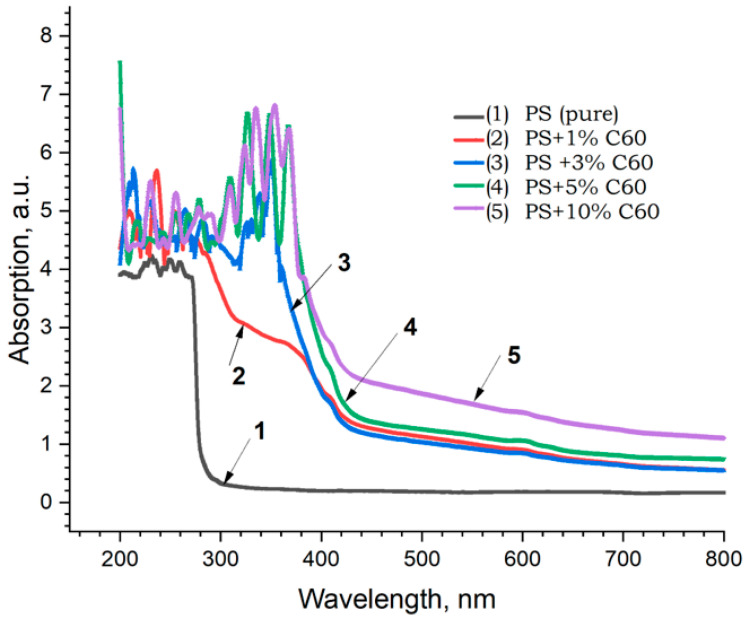
Absorption spectra of PS + Fullerene C60 samples: 1—PS pure; 2—PS + 1% C_60_; 3—PS + 3% C_60_; 4—PS + 5% C_60_; 5—PS + 10% C_60_.

**Figure 15:**
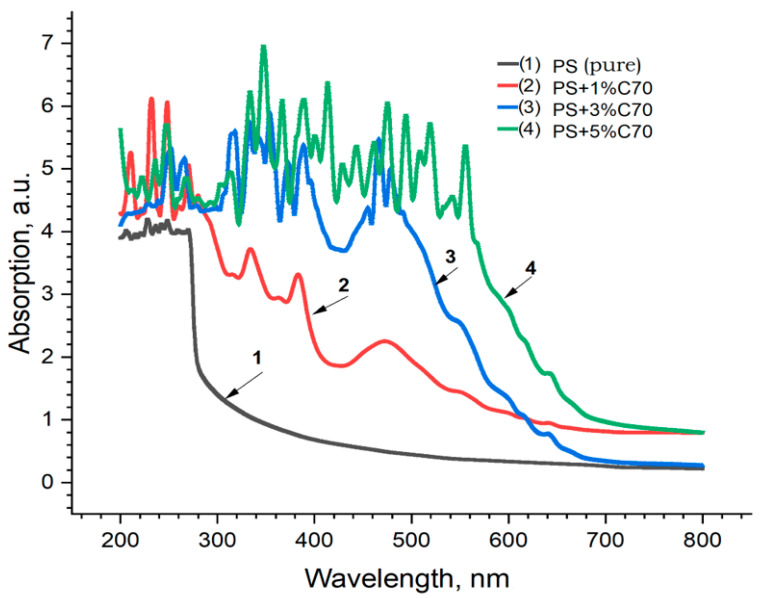
Absorption spectra of PS + Fullerene C_70_ samples: 1—PS pure; 2—PS + 1% C_70_; 3—PS + 3% C_70_; 4—PS + 5% C_70_.

In the absorption spectra of the samples, the main changes are observed in the UV range (190–300 nm) of wavelengths, which are also confirmed in [37] inset in Figure 14. In general, with increasing concentration of carbon nanoparticles (MWCNTs) in all the spectra, a tendency of increasing absorption capacity towards the short wavelength region and correspondingly decreasing reflectance and transmittance is observed. Similar results were also obtained for the samples of PS + fullerene C_60_ and PS + fullerene C_70_ groups (Figure 14 and Figure 15).

As can be seen, the absorption spectra of all the studied samples show an increase in the absorption coefficient as the concentration of carbon nanoparticles increases. Adding carbon nanoparticles to the matrices of polymer films does not produce any changes to the chemical composition of the polymer, but in combination with the polymer matrix forming UNP films, a number of their physical properties are significantly affected.

**Figure 16:**
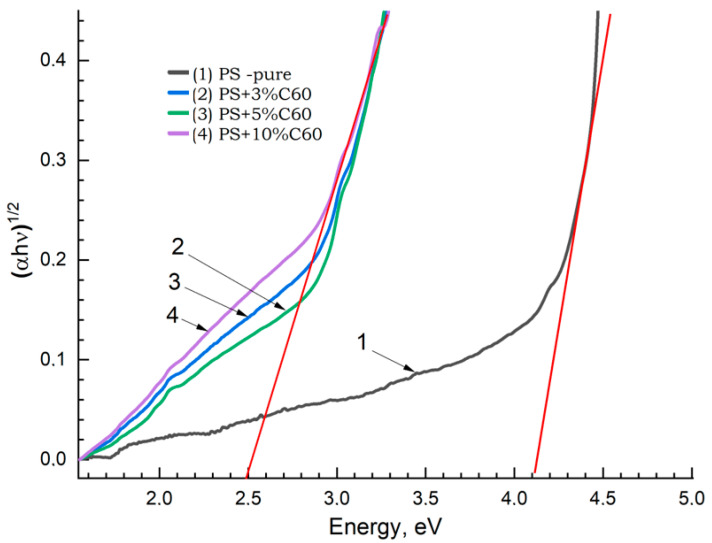
Results of the concentration dependence of the band gap energy for PS + C_60_ samples: (1) PS pure; (2) 3%; (3) 5%; (4) 10%.

According to the dependence graph (αE_F_)^1/2^ = f(E_F_) for samples of the PS + C_60_ group, by extrapolating a linear section to the abscissa axis E_g_ along the intersection point, the energy of the band gap was determined. The results of this calculation are shown in Figure 16. As can be seen from the figures, when going from a pure PS sample with a band gap energy E_g_ of more than 4.1 eV to a sample containing 10% C_60_ fullerene, Eg decreases to ≈2.5 eV.

The obtained results confirm that, in terms of the value of units, with an increase in the concentration of nanoparticles, the studied samples move from values characteristic of dielectric materials (E_g_ > 3 eV) to semiconductor materials (E_g_ < 3 eV). The results of such a calculation are shown in Figure 16.

Studies show that the width of the forbidden zone decreases significantly. At a concentration of 5% C_60_, the smallest width is observed. Likely, a decrease in the band gap of the studied samples with an increase in the concentration of carbon nanoparticles may be associated with an increase in their structural disorder. For a pure PS sample, the obtained E_g_ values are confirmed by data in the literature; for example, [37,38].

We can illustrate this phenomenon using a specific study where the calculated energy gaps of solid composite samples demonstrate that the electronic transition from the valence band to the conduction band is direct in nature. When Cu was added, an increase from 1.2 to 2.1 of the refractive index was observed, and a decreased value of the energy gap from 4.05 to about 3.65 eV took place as a result of the presence of multiple states. The observed low refractive index values for PS-based composites show the significance of their waveguide applications. The fact that the energy gap and the refractive index are strongly correlated can be confirmed by the results of this study [39].

There is also known work where it was observed that the direct energy gap related to pure PS decreased from the value of 4.0 eV to 2.0 eV when substituted with a PS composite thin film, and the indirect energy gap decreased from 3.8 to 2.95 eV after CNT and CN filling, respectively. The Urbach energy increased from 2.120 to 4.583 eV after CNT and CN were added to the PS matrix. The average roughness and RMS value of roughness increased from 1.94 and 2.58 nm for the pure material to 2.34 and 3.22 nm, 2.16 and 2.71, and 3.16 and 4.24 for PS-0.05CNT, PS-0.05CN, and PS-0.025CNT-0.025CN composite thin films, respectively [40].

In a similar study, the irradiation of PS/CuONPs by Nd-YAG resulted in a consistent change in various sample properties, such as chemical, optical, and compositional [41]. The chemical resistance of the PS by the Nd-YAG irradiation shows the carbonaceous material formed based on the chemical properties of FTIR. The energy band gap obtained by the Tauc equation proved that the energy band gap decreased with the increase (from 4.29 eV to 1.38 eV) of time bombardment and the concentration of the copper oxide nanoparticles.

It is known that polymer doping with carbon-based nanoparticles (fullerenes, nanotubes, nanodiamond particles, and others) changes several physical, chemical, and operational characteristics of the original polymer materials. Depending on various factors of interactions between the matrix and carbon nanoadducts, these changes can lead to both improvement and deterioration of physical characteristics. In the framework of the present work, some optical properties of carbon composite polymeric materials based on polystyrene have been experimentally investigated, and their bandgap energies have been determined [42].

## 4. Conclusions

The problems of some waste disposal from Kazakhstan can be solved positively because they are safe and contain micro- and nanoparticles. Their use in various composite materials, in this case in polymer compositions, has a very positive effect on technological and operational characteristics: increased corrosion resistance, toughness, and strength characteristics.

Polymers that are contained in industrial waste can be recycled by adding nano-additives to them. In this case, the physical properties also change, which opens up new opportunities to improve the performance of semiconductors and other materials. The experimental results show that the optimal additive content is 2% (by weight) of microsilica and 5% (by weight) of microspheres, as they produce improved physical and mechanical properties of epoxy composites in comparison with the standard (control) material. Studies have shown that the introduction of 5% microspheres into ED-20 polymer increases the impact strength by 9%. As a result of the modification of the composite structure, the presence of 30–300 µm microsphere particles showed higher impact strength results. The introduction of microsilica in the matrix in the amount of 2% contributes to increasing the modulus of elasticity by 19%. These experiments indicate the potential for compositional variability and the development of modification technology.

Polymers contained in municipal solid waste can be recycled and reused by adding nano-additives to them. This changes the physical properties, which opens up new opportunities for improving the performance of semiconductors and other materials. Spectrophotometric studies of carbon-containing composite polymer films based on polystyrene were carried out. Absorption spectra (reflection and transmission) of samples with the addition of different concentrations of three types of carbon nanoparticles—fullerenes C_60_, C_70,_ and MWCNTs—were obtained. It has been shown that in the absorption spectra of all studied samples, an increased coefficient of absorption can be observed with increasing concentration of nanoparticles. The band gap energy of the investigated samples was established based on the concentration of added nanoparticles. It was discovered that as the concentration of nanoparticles increases, the energy value of the samples’ band gap decreases; in particular, this indicator for the PS + C_60_ sample decreases in the range from 4.1 eV (for pure PS) to ≈2.5 eV (for the PS + 10% C_60_ sample). In PS, fullerene particles, due to weak interaction with polymer macromolecules (reaction between benzene pendants of the polymer and C_60_ hexagons), aggregate into relatively large clusters, which, in turn, can lead to loosening of the polymer matrix, the formation of many defects such as micropores and microcracks, and a decrease in the integral strength and deformability.

It should also be noted that with increasing nanoparticle concentration, the studied samples go from values characteristic of dielectric materials, Eg > 3 eV, to semiconducting materials where Eg < 3 eV.

Studies indicate that the degree of interaction between the polymer and fillers is crucial in the mechanical behavior of composites. It is noted that the primary industrial method of the production of polymer composites based on the base polymer from solid domestic waste is the mechanical mixing of filler and polymer matrix. These materials offer a synergistic combination of the characteristics of their components, resulting in improved mechanical, thermal, electrical, and other functional properties.

## Figures and Tables

**Table 1:** The studied samples: carbon composite polymer films based on PS.

No.	Samples	Mass Concentration, wt%	Sample Thickness, µm
1	Polystyrene (pure)	0	30
2	Polystyrene + MWCNT	0.1	85
		0.5	95
3	Polystyrene + Fullerene C60	1	50
		3	45
		5	105
		10	110
4	Polystyrene + Fullerene C70	1	50
		3	65
		5	90

**Table 2:** The microsphere and microsilica elemental composition.

Chemical Element	Microsphere, %	Microsilica, %
Na	0.133	0.098
Al	11.458	15.239
Si	25.312	21.485
P	0.117	0.235
K	0.690	0.425
Ca	1.798	2.265
Ti	0.815	0.752
V	2.200	1.780
Mn	3.450	2.990
Fe	3.270	4.110
Sr	6.260	5.120
Zr	3.780	2.220

**Table 3:** Strength and tensile strength of polymer compositions with additives.

Composition	Tensile Strength, MPa	Tensile Modulus of Elasticity, MPa
ED-20 + PEPA	19.0	1598
Microsphere 1%	19.1	1683
Microsphere 2%	19.2	1867
Microsphere 5%	23.9	1954
Microsphere 10%	15.8	1932
Microsphere 15%	16.9	2011
Microsphere 20%	19.7	2101
Microsilica 1%	22.4	1805
Microsilica 2%	27.6	1702
Microsilica 5%	18.7	1321
Microsilica 10%	12.9	989
Microsilica 15%	13.7	1402
Microsilica 20%	12.1	1305

**Table 4:** Impact toughness of composite materials.

Composition	Impact Energy, J	Impact Strength, J/cm^2^
ED-20 + PEPA	0.191	0.444
ED + Microsphere 1%	0.242	0.452
ED + Microsphere 2%	0.225	0.678
ED + Microsphere 5%	0.206	0.652
ED + Microsphere 10%	5.325	16.398
ED + Microsphere 15%	5.201	16.207
ED + Microsphere 20%	5.403	16.302
ED + Microsilica 1%	0.298	1.085
ED + Microsilica 2%	0.375	1.224
ED + Microsilica 5%	0.219	0.696
ED + Microsilica 10%	0.258	0.819
ED + Microsilica 15%	0.201	0.621
ED + Microsilica 20%	0.198	0.602

## Data Availability

The data presented in this study are available upon request from the corresponding author.
